# Metal Biomonitoring Through Arboreal Species in Riparian Ecosystems: *Pithecellobium dulce* as a Bioindicator Species

**DOI:** 10.3390/plants14010118

**Published:** 2025-01-03

**Authors:** Sayuri Hernández-Maravilla, María Luisa Castrejón-Godínez, Efraín Tovar-Sánchez, Hugo Albeiro Saldarriaga-Noreña, Alexis Rodríguez, Marcos Eduardo Rosas-Ramírez, Patricia Mussali-Galante

**Affiliations:** 1 Laboratorio de Investigaciones Ambientales, Centro de Investigación en Biotecnología, Universidad Autónoma del Estado de Morelos, Av. Universidad, 1001, Col. Chamilpa, Cuernavaca C.P. 62209, Morelos, Mexico; sayu.maravilla@gmail.com (S.H.-M.); alexis.rodriguez@uaem.mx (A.R.); marcos.rosas@uaem.edu.mx (M.E.R.-R.); 2 Facultad de Ciencias Biológicas, Universidad Autónoma del Estado de Morelos, Av. Universidad, 1001, Col. Chamilpa, Cuernavaca C.P. 62209, Morelos, Mexico; 3 Centro de Investigación en Biodiversidad y Conservación, Universidad Autónoma del Estado de Morelos, Av. Universidad, 1001, Col. Chamilpa, Cuernavaca C.P. 62209, Morelos, Mexico; efrain_tovar@uaem.mx; 4 Centro de Investigaciones Químicas, Universidad Autónoma del Estado de Morelos, Av. Universidad, 1001, Col. Chamilpa, Cuernavaca C.P. 62209, Morelos, Mexico; hsaldarriaga@uaem.mx

**Keywords:** water pollution, metals, bioindicator species, *Pithecellobium dulce*, riparian ecosystems

## Abstract

Water pollution by metals is a global environmental problem. In riparian ecosystems, metal pollution generates adverse effects on organisms and reduces water quality. The Cuautla River is of great ecological relevance and an important water supplier. However, it is polluted by multiple wastewater discharges from different origins, with toxic metals being the main pollutants. Therefore, environmental monitoring strategies based on bioindicator species are necessary to evaluate the ecosystem health of riparian ecosystems. *Pithecellobium dulce* (Roxb.) Benth is a tree species native to Mexico, widely distributed including in riparian ecosystems, and it is also established in contaminated sites. In this study, Cd, Cu, Pb, and Zn concentrations in water and sediment and in leaf and bark of adult *P. dulce* trees established in six sampling sites along the Cuautla’s riverbed were determined. Likewise, the genotoxic damage derived from metal exposure to leaves was evaluated. The results evidenced the presence of Cd and Pb in water and Cd, Cu, Pb, and Zn in sediment. *P. dulce* registered high levels of Cu, Pb, and Zn in both leaf and bark, showing higher concentrations in leaf than in bark. In addition, the greater the concentration of Pb in leaves, the greater the genotoxic damage observed, while the concentration of Cu and Zn did not show a relationship with the genotoxic damage in leaves. Overall, Cu and Pb concentrations in leaves enabled us to detect pollution gradients for these metals in water and sediment from the sampling sites. Due to its wide geographic distribution, establishment in polluted sites, and metal absorption capacity, *P. dulce* can be considered a bioindicator species for environmental health studies in riparian ecosystems contaminated with metals.

## 1. Introduction

Anthropogenic activities such as diverse industries, agriculture, livestock, mining, and urbanization generate the release of metals into the environment [1]. Metals can bioaccumulate in different organisms and may biomagnify through food chains [2], generating important ecotoxicological risks in terrestrial, aquatic, and riparian ecosystems.

Riparian ecosystems are transition zones between aquatic and terrestrial ecosystems [3] that provide essential ecosystem services such as habitat for a great number of species [4], biomass production for fuel, regulation of physicochemical and biological conditions, pollination and seed dispersal [5], carbon sequestration [6], and erosion stabilization and control [7], among others. Likewise, riparian vegetation supports biogeochemical cycles [8], regulates the speed of the river channel, the infiltration and storage of water in the subsoil [9], and controls temperature, light, and the regulation of microclimates [10].

However, riparian ecosystems have been threatened [11] by various anthropogenic activities, where metals are among the main pollutants. When bioavailable, these elements may be absorbed by plants, which enter through their root system [12], causing cytotoxicity and genotoxicity [13] by interfering with different key metabolic pathways [14]. Metal bioaccumulation promotes the production of reactive oxygen species (ROS), inducing the oxidation of essential macromolecules such as DNA [15], altering the genetic repair mechanisms, causing mutations, single- and double-stranded DNA breaks, and adduct formation, as well as the generation of chromosomal aberrations and micronuclei [16].

Despite this, many plant species can tolerate and settle in environments with high metal concentrations, since they have efficient detoxification mechanisms [17], such as sequestration, chelation, and exclusion [18]; certain plant species are even known as “metal hyperaccumulators” which are capable of bioaccumulating high metal concentrations in their tissues [19]. Metal accumulator plants can be used for phytoremediation of contaminated sites [20]; moreover, some of these plants are considered as bioindicators of environmental quality [21,22,23] due to their wide geographical distribution, great abundance, sensitivity to environmental stressors, non-lethal biological effects induced by exposure to pollutants, and their capacity for pollutant accumulation [24].

Particularly, due to their longevity, trees located in polluted sites may be chronically exposed to metals and considered as environmental bioindicators [25]. Moreover, analyzing metal content in tissues such as bark enables us to collect data on long-term contamination, using it as a biomonitor for environmental pollution. Also, bark is widely available without affecting tree health and it can be exposed to pollution either directly from the atmosphere or from stem flow. Its structural porosity makes bark an efficient tissue for accumulation and metal determination [26].

Some tree species have been used as biomonitors, like *Salix* ssp., *Junglans regia* (Juglandaceae) and *Populus nigra* (Salicaceae) [27,28], *Pinus halepensis* (Pinaceae) [29], *Prosopis laevigata* (Fabaceae) [30], and *Pithecellobium dulce* (Fabaceae) [31]. Specifically, *P. dulce* is a native to Mexico, with wide geographic distribution and drought tolerance [32,33,34]. *P. dulce* has been studied to monitor atmospheric metal pollution [35], for its ability to remove Pb (II) from water [36], and its Cr phytoremediation potential [37]. Therefore, in this study, we determined whether *P. dulce* can be used as a bioindicator species of environmental health in riparian ecosystems by analyzing the metal content in leaf and bark tissues and the genotoxic effect in leaves of trees growing along the Cuautla River, and if metal concentration in leaves can be explained by their concentrations in water and sediment at the study sites along the riverbed.

## 2. Results

### 2.1. Metal Concentrations in Water Samples from the Cuautla River

Metal concentrations determined in water samples from the six sampling sites in the Cuautla River were compared with the permissible limits established by the Mexican official standard NOM-127-SSA1-2021 [38] and the water quality standards established by the World Health Organization (WHO) [39] (Table 1). In general, Pb was detected in all the sites; in contrast, Cd was only detected in S1. In S1, Cd concentration in water exceeded the maximum permissible limits set by the national (1.2 times) and international (2.0 times) standards. From S1 to S6, Pb concentrations in water exceeded the limits established for both national and internationals standards: 50.9 times for S1, 13.6 times for S2, 15.0 times for S3, 14.1 times for S4, 31.1 times for S5, and 10.2 times for S6.

### 2.2. Metal Concentration in Sediment Samples from the Cuautla River

Since there are no standards that regulate the maximum permissible limits of metals in sediments, the obtained values in the sediment samples from the Cuautla River were compared with various international standards like the Canadian Council of Ministers of the Environment [40,41,42,43] through the sediment quality guidelines for the protection of aquatic life (ISQG) and the levels with probable effects (PEL).

Metal concentration in sediments from the six sampling sites in the Cuautla River are shown in Table 2. In general, Cr and Ni were not detected in sediment samples. In S1, Cu, Cd, Pb, and Zn were detected; however, in sites S2, S3, S4, S5, and S6, only Pb and Zn were detected. With respect to ISQG values established by the Canadian Council of Ministers of the Environment, Cu concentration was 1.3 times higher, Cd 149.2 times higher, and Pb 12.2 times higher, while the concentration of Zn was below the established limits. Regarding the maximum permissible values with probable effects (PEL), Cd and Pb exceeded these values by 25.6 and 4.7 times, respectively, in S1. From S2 to S6, only Pb and Zn were detected, while Pb concentration in sediments from S2, S3, and S4 exceeded 1.6 times the values of the maximum limits established in the ISQG.

### 2.3. Relationship Between Metal Concentrations in Water and Sediments and the Sampling Distance from the S1

Linear regressions were performed to determine the relationship between the sampling site distances starting from S1 (0 m) and Cd, Cu, Pb, and Zn concentrations in water and sediment. In water, only Cd and Pb were detected. In contrast, all analyzed metals were registered in sediment samples. A negative and significant relationship was documented between the sampling site distance and Cd, Cu, and Pb concentration in water and sediment; in other words, as the distance from S1 increases, the metal concentration in water and sediment decreases. Regarding Zn, a positive and significant relationship was observed between Zn concentration in sediment and the sampling site distance, since the greater the distance from S1, the higher the concentration of Zn in the sediment.

**Figure 1:**
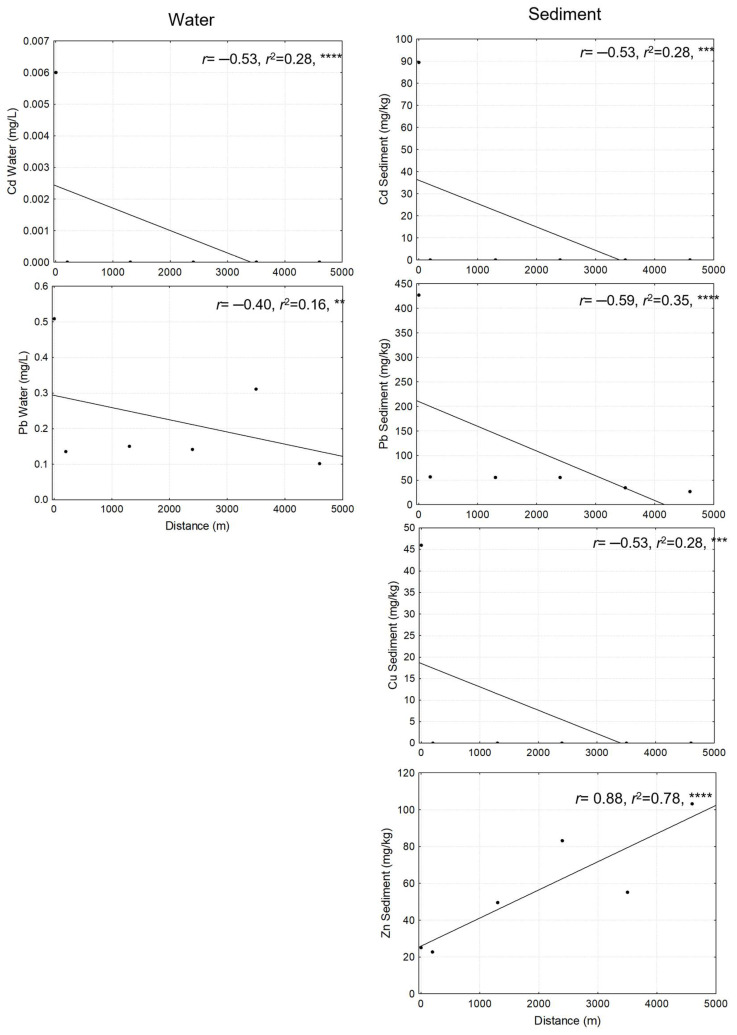
Regression analysis between metal concentration in water and sediments and the sampling site distance from the S1 (0 m). ** = *p* < 0.01, *** = *p* < 0.001, **** = *p* < 0.0001. Regression analyses for Zn and Cu are not shown because these metals were not detected in water.

Based on our results, a pollution gradient of Pb and Cd was identified along the sampled sites at the Cuautla River. Specifically, the highest levels of Pb and Cd in water were obtained in S1 (0 m). In sediment, a similar pollution gradient was found, showing the highest Cd, Cu, and Pb concentrations in S1 (0 m). In contrast, the highest Zn concentration was registered in S6 (5000 m from S1) (Figure 1).

### 2.4. Metal Concentrations in Leaf and Bark Tissue of P. dulce

In general, we registered the accumulation of Cu, Pb, and Zn in leaf and bark tissues of *P. dulce*. In contrast, Cd, Ni, and Cr were not detected. In foliar tissue, Cu concentrations ranged from 25.4 mg/kg (S2) to 44.6 mg/kg (S6), Pb from 62.8 mg/kg (S1) to 7.9 mg/kg (S6), and Zn from 27.7 mg/kg (S2) to 3.2 mg/kg (S6). In bark tissue, Cu concentrations ranged from 13.4 mg/kg (S5) to 2.3 mg/kg (S6), Pb from 37.6 mg/kg (S1) to 11.9 mg/kg (S5), and Zn from 15.7 mg/kg (S5) to 9.1 mg/kg (S3). All the analyzed individuals presented greater Pb concentrations than Cu and Zn, except for individuals at S5, where a higher Zn concentration was found (Table 3).

### 2.5. Relationship Between Metal Concentrations in Leaf and Bark Tissue of P. dulce and Metal Concentration in Water and Sediment of the Cuautla River

**Figure 2:**
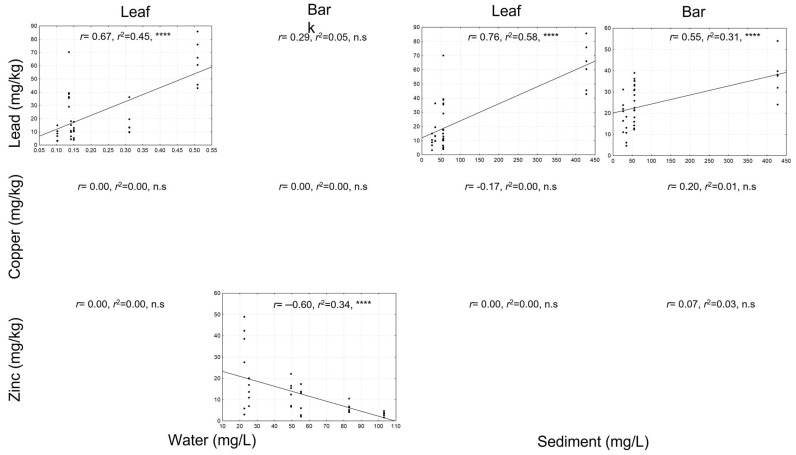
Regression analysis between metal concentration in leaf tissue and bark of *P. dulce* and metal concentration in water and sediment. n.s = no significant differences (*p* > 0.05). **** = *p* < 0.0001. Only correlations significant at *p* < 0.05 are shown.

Linear regressions were performed to determine the relationship between Pb, Cu, and Zn concentrations in leaf and bark tissues of *P. dulce* and metal concentrations in the water and sediment of the Cuautla River (Figure 2). Positive and significant relationships were registered between Pb concentration in leaves, water, and sediment. Likewise, a positive and significant relationship was identified between Pb concentrations in bark and in sediments. However, no relationship was identified between Pb concentration in bark and in water. For Zn, only a negative and significant relationship was registered between Zn concentration in bark and in water, while no relationship was identified between Zn concentration in bark and in sediment. For Cu, no relationship was identified between its concentration in leaf and bark tissue and in environmental matrices.

### 2.6. Relationship Between Metal Concentration in Leaf and Bark Tissues of P. dulce and Sampling Distance

**Figure 3:**
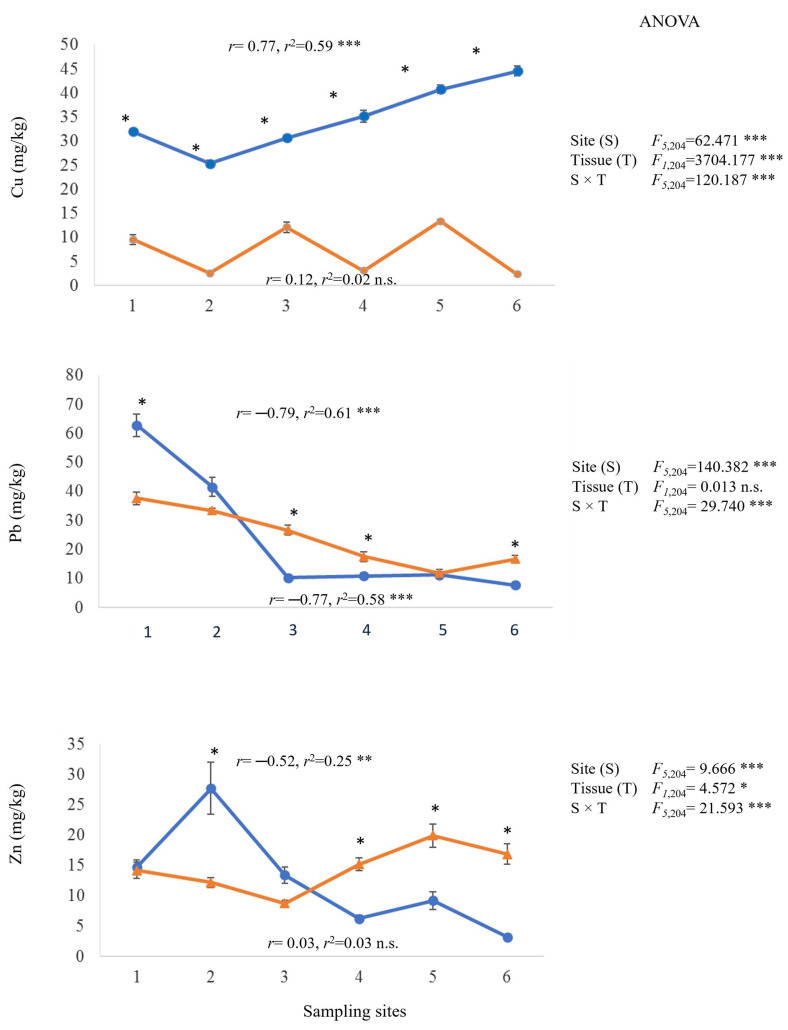
Metal concentration (average ± standard error), two-way ANOVA to evaluate the effect of site, tissue (leaf and bark), and interaction (site × tissue) in *P. dulce*. Regression analysis between sample sites and metal concentration in leaf (blue lines) and bark (orange lines) tissues. The asterisks denote significant differences between tissues by exposure site with *p* < 0.05 (Tukey). ANOVA test: *** = *p* < 0.001, ** = *p* < 0.01, * = *p* < 0.05, n.s. = no significant differences.

Linear regression analyses were performed to determine the relationship between Cu, Pb, and Zn concentrations in leaf and bark tissues of *P. dulce* and the sampling site distances from S1 (0m). The results showed a negative and significant relationship between Pb concentration in leaves and bark and the sampling site distances from S1 (Figure 3). Likewise, a Pb pollution gradient was identified, since the greater the distance from S1, the lower the Pb levels in both tissues.

**Figure 4:**
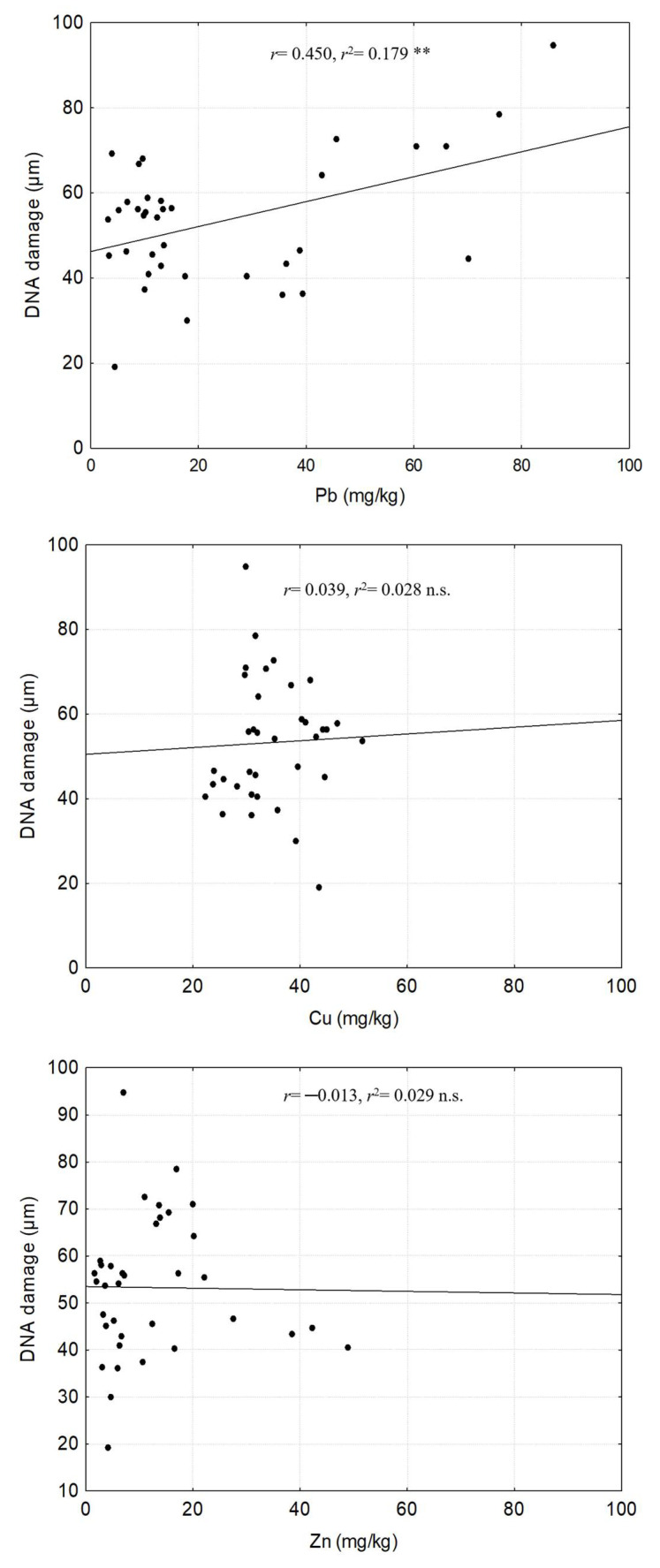
Relationship between metal concentration in leaf tissue of *P. dulce* and genotoxic damage. The graphs show the linear regressions to determine the relationship between Pb, Cu, and Zn concentration in leaves and the observed genotoxic damage. The values of the linear regressions are indicated at the top of each graph. n.s. = not significant, ** = *p* < 0.01.

A positive and significant relationship was identified between Cu concentration in leaves and the sampling site distances, since the greater the distance from S1, the greater the registered Cu levels, indicating a Cu pollution gradient. In contrast, no relationship between Cu concentrations in bark and the sampling site distances was registered (Figure 4). For Zn, a negative and significant relationship was identified between Zn concentrations in leaves and the sampling site distances, and no relationship was observed between Zn content in bark and the sampling distance (Figure 3).

The results of the two-way analysis of variance (ANOVA) showed that the site (S), the tissue (T), and the interaction (S × T) had a significant effect on Cu, Pb, and Zn concentrations in *P. dulce*, except for Pb concentration between tissues (Figure 3).

### 2.7. Relationship Between Metal Concentration in Leaves and in Bark Tissue of P. dulce

Linear regression analyses were performed to determine the relationship between metal concentration (Pb, Cu, and Zn) in leaves and bark of *P. dulce*. A positive and significant relationship was found between Pb concentration in leaves and in bark (r = 0.520, r^2^ = 0.25, *p* < 0.0001). In contrast, no relationship was observed between Cu and Zn content in leaves and in bark (Cu: r = 0.08, r^2^ = 0.03, *p* = 0.96; Zn: r = −0.13, r^2^ = 0.01, *p* = 0.46).

Also, the relationship between the levels of essential (Cu and Zn) and non-essential metals (Cd and Pb) in foliar tissue of *P. dulce* was evaluated. A positive and significant relationship was found between Pb and Zn concentration in leaves (r = 0.400, r^2^ = 0.14, *p* = 0.01). In contrast, a negative and significant relationship was found between Pb and Cu concentration in leaves (r = −0.49, r^2^ = 0.22, *p* = 0.002).

### 2.8. Relationship Between Metal Concentration in Leaves and Genetic Damage in P. dulce

A positive and significant relationship was found between Pb concentrations in *P. dulce* leaves and the genetic damage (DNA single-strand breaks) observed. On the contrary, no relationship was documented between Cu and Zn concentration in leaves and the genetic damage observed in *P. dulce* individuals (Figure 4).

## 3. Discussion

### 3.1. Metal Concentrations in Water and Sediment Samples from the Cuautla River

In the present study, Cd (0.006 mg/L) and Pb (0.102–0.509 mg/L) concentrations were identified in water samples from the Cuautla River. Lead concentrations exceeded all the maximum permissible limits established by different regulatory organizations corresponding to water for human use and consumption, (WHO; Pb: 0.01 mg/L), and the Mexican official standard NOM-127-SSA1-2021 [38] (Pb: 0.01 mg/L).

According to metal concentrations in sediment samples, we identified that S1 is a source of Cd and Pb contamination. The presence of these metals in the Cuautla River may be related to industrial activities, mainly the soft drink bottling and automotive industries, along with industrial, domestic, and hospital wastewater discharges, and farming and livestock activities. Particularly, the contribution of soft drink bottling plants to water and sediment pollution has been described, mainly in the process of the exhaustive washing of the bottles [44], that wears away the paint on the labels, rich in metals such as Cr, Cd, and Pb, that can reach water bodies in high concentrations, as reported by Singh et al. [45]: Cd (1.0–35.9 mg/kg), Cr (21.8–265.3 mg/kg), Hg (12.1–132.3 mg/kg), Ni (8.9–143.4 mg/kg), Pb (15.4–362.4 mg/kg), and As (0.6–49.7 mg/kg). Also, the release of metals (mg/L) from automotive paint has been reported [46], among which are Cd (No detected-1.44 µg/m^3^), Cr (0.02–4.46 µg/m^3^) and Pb (No detected-26.34 µg/m^3^) [47]. Furthermore, concentrations of Pb and Cd higher than the permissible limits set by the NOM-127-SSA1-2021 have been reported in water wells and springs derived from the infiltration of contaminated surface water [48].

Lead concentration in sediment registered a positive and significant relationship with Pb concentration in leaves and bark of *P. dulce*; this could be due to the higher bioavailable Pb concentration in sediment, where the primarily bioavailable Pb fraction could be found in the form of carbonates, oxides, or in complexes with organic matter [49]. In contrast, only a positive relationship was identified between Zn concentration in water and Zn in leaves, a fact that may be explained since *P. dulce* roots are anchored to the sediment, where a greater absorption of metals from it can be reached. Moreover, it has been reported that, when Pb and Zn are bioavailable in the same environmental matrix, a competition between them can happen [50].

Similar studies carried out in riparian ecosystems contaminated with metals in Morelos State (Yautepec River) evaluated 10 water samples, in which the presence of Cu, Cd, Cr, Ni, Pb, Zn, Mn, and Fe were identified, highlighting the presence of Pb (0.0093–0.0808 mg/L) and Cd (0.0002–0.0095 mg/L). The authors report that the presence of Cd and Pb may be due to industrial wastewater, from pharmaceutical, cosmetic, dye, automotive, food, chemical, agrochemical, metallurgical, and textile industries [51]. It should be noted that the Yautepec River is located near the Cuautla River, where high concentrations of Cd and Pb were also detected.

There are several examples in the literature which document water and sediment contamination due to industrial activities that increase the presence of metals. For example, the Atoyac River (Tlaxcala, Mexico) presented high levels (mg/L) of Al, Cu, Cr, Fe, Pb, and Zn related to agricultural and industrial activities such as galvanization, paints, ferroalloys, and discharge of urban and industrial wastewater [52]. Also, Mancilla-Villa et al. [53] detected high levels of Pb (0.0015 mg/L) in the Ayuquila River (Jalisco and Colima, Mexico) that exceeded the permissible limits established by the NOM-001-SEMARNAT-2021 [54], indicating that Pb was discharged into the river through wastewater and improper disposal of batteries, electronic waste, paints, welding materials, and fuels. These findings coincide with the study of Ali et al. [55], who reported high Cd (0.001 mg/L; 1.67 mg/kg) and Pb (0.024 mg/L; 25.45 mg/kg) concentrations in water and in sediment, respectively, in the Bhairab River (Bangladesh), where mixed sources of anthropogenic pollution such as industrial effluents and agricultural activities are the cause of the pollution.

The results obtained in the present study agree with the previous examples of the origin of anthropogenic contamination and the main industrial activities that generate metal pollution in water and sediments near these pollution sources, which put human and environmental health at risk. This demonstrates the need to conduct continuous environmental biomonitoring studies for these sites.

### 3.2. Metal Concentrations in P. dulce

In this study, *P. dulce* showed concentrations levels up to 44.6 mg/kg for Cu, 62.8 mg/kg for Pb, and 27.7 mg/kg for Zn in leaves. It has been documented that *P. dulce* can accumulate Cu (28.88 mg/kg), Pb (14.71 mg/kg), and Zn (54.19 mg/kg) in shoots [31]; likewise, trees of the Fabaceae family such as Cassia fistula have been reported to bioaccumulate Cu (26.33 mg/kg), Pb (15.56 mg/kg), and Zn (65.67 mg/kg) in shoots [31], as well as Prosopis laevigata, which accumulates Cu (0.93 mg/kg), Pb (1.62 mg/kg), and Zn (0.65 mg/kg) in leaves [30]. Also, Santoyo-Martínez et al. [20] reported Cu (0.38 mg/kg), Pb (4.75 mg/kg), and Zn (0.21 mg/kg) accumulation in Vachellia campechiana leaves after 360 days growing on a substrate with mining waste under greenhouse conditions. Also, Eid et al. [56] reported Cu (8.49 mg/kg), Pb (6.03 mg/kg), and Zn (39.2 mg/kg) concentrations in Pisum sativum grown in agricultural soils supplemented with a mixture of residual sludge. Hence, when comparing the results obtained in the present study with those mentioned above, and because *P. dulce* showed higher Cu and Pb concentrations, *P. dulce* represents a tree species with the potential to phytoremediate riparian ecosystems polluted with Cu and Pb. The present study found that *P. dulce* in the Cuautla River presents higher Pb concentrations in leaves than the studies reported in the literature for species of the Fabaceae family. It should be noted that this is the first study where *P. dulce* bark tissue is used as a valuable tissue to study chronic exposure to metals present in water and sediments.

Regarding metal content in *P. dulce* bark, Cu (13.4 mg/kg), Pb (37.6 mg/kg), and Zn (15.7 mg/kg) concentrations were detected, which indicates a greater metal content in leaves than in bark. The high Cu concentrations detected in leaves could be explained by the highly dynamic photosynthetic nature of the leaves, where Cu is a micronutrient that can be mainly redirected to this tissue, acting as a cofactor for numerous Cu-proteins [57], among which superoxide dismutase and cytochrome C oxidase stand out [58], which perform important functions in plant cells, such as electron transport and chloroplast and mitochondrial homeostasis [59]. Moreover, Cu participates in hormonal signaling by binding to Cu-dependent phytohormone receptors in cell wall metabolism, carbon assimilation, ATP synthesis, and plant growth, as well as in response to chemical stress [60].

Regarding Pb concentration, no significant differences were determined between leaves and bark, meaning that both tissues present this metal in similar concentrations. When Pb is absorbed and then translocated to the stem by passive transport through the H^+^/ATPase system, most Pb^2+^ ions are first translocated apoplastically through the endodermis and subsequently transported simplistically by the vascular tissues [19]. Most of the Pb that is internalized into the plant is sequestered in the cell wall and the vacuoles [61]; however, when significant amounts of Pb are internalized, they cause an imbalance in the metal barrier system of the plasmalemma and the tonoplast, causing damage to the cell and allowing the passage of Pb to various plant tissues without distinction [62]. It has been reported that the Pb toxic range for plants is from 10 to 30 mg/kg [63], while, in the present work, it was found that *P. dulce* individuals show concentrations that range from 7.9 ± 1.8 to 62.8 ± 6.9 mg/kg in leaf tissue, indicating that *P. dulce* is a tolerant species to high Pb concentrations, which supports the idea of its usefulness as an indicator species for Pb contamination and its possible use in phytoremediation strategies.

Zinc content in leaves ranged from 3.2 ± 0.4 to 27.7 ± 7.9 mg/kg. Zinc is a micronutrient for plants; it plays a fundamental role in the biosynthesis and functioning of different enzymes, such as auxins and regulatory proteins of the Zn finger type, as well as in the production of chlorophyll [14]. Zn is required to maintain the integrity of the ribosomes and for the correct functioning of RNA polymerase, and it also plays an important role in gene transcription [64]. It has been reported that Pb bioaccumulation interferes with the uptake of essential metals such as Zn and Cu by inhibiting nutrient adsorption mechanisms in the roots and blocking the entry of cations [61]. Zinc deficiency has been observed when its concentration ranges from 15 to 20 mg/kg [63], causing chlorosis and delayed plant growth.

When assessing bioaccumulation processes and metal content in trees, bark is also a useful tissue because it can accumulate high metal concentrations; metallic ions remain attached to the cell wall through adsorption mechanisms [65], serving as a storage tissue which, in turn, can be effective as a detoxification mechanism. Bark allows for the monitoring of pollution for long periods as it is a lasting tissue, bringing evidence of chronic metal exposure. For example, Sevik et al. [66] evaluated air pollution by heavy metals from 1978 to 2016 in Kastamonu (Turkey), reporting fluctuations in pollution over time through the bark rings of the *Cedrus* sp. (Pinaceae) tree.

We identified greater Pb concentrations in the individuals established in sampling sites S1 and S2, since the source of Pb contamination is probably closer to S1. On the other hand, individuals from sampling sites S4, S5, and S6 showed the same concentration pattern Cu > Pb > Zn, that is, the source of Cu contamination is closer to S6. According to Kaur et al. [67], *P. dulce* is considered an accumulator plant of Pb in different tissues (root > bark > stem > leaf > fruit) when grown on mining tailings. Likewise, Qadir et al. [31] reported that *P. dulce* accumulates various metals Cd > Fe > Zn > Cr > Ni > Cu > Mn > Pb in leaf tissue when it is grown on soils contaminated with fly ash. Other species of the Fabaceae family such as Prosopis laevigata established in mine tailings can also bioaccumulate Cu, Fe, Pb, and Zn in roots and leaf tissue [30], as well as Vachellia campechiana, which bioaccumulates Pb > Fe > Cr > Cu > Zn in roots and leaf tissue [20]. Also, Trifolium alexandrinum established on agricultural soils contaminated with metals bioaccumulates Cr, Cu, Cd, Co, and Pb [68]. A study conducted with five tree species (Albizia lebbeck, Bauhinia purpurea, Dalbergia sissoo, Millettia peguensis, and Pongamia pinnata) of the Fabaceae family, irrigated with untreated industrial wastewater, showed the bioaccumulation of Cr, Cu, Mn, and Pb in root, stem, and leaf tissues, translocating Pb to the leaf [69]. All the species registered Pb and mostly Cu and Zn; hence, the present study supports the idea that *P. dulce* is capable of absorbing metals, which is useful for phytoremediation strategies. Furthermore, assessing the metal content in leaves and bark of tree species is informative to infer time of exposure to these contaminants and allows for constant and long-term biomonitoring that indicates acute exposure (leaf tissue) and chronic exposure (bark) to metals.

### 3.3. Genetic Damage in Individuals of P. dulce Established in the Cuautla River

The alkaline single-cell electrophoresis technique or “Comet assay” allows for the visualization of genetic damage such as single-strand breaks (SSB), alkali-labile sites, and delayed repair sites. In the present study, a positive and significant relationship was identified between the Pb concentration and genetic damage in *P. dulce* leaves; the individuals with the highest Pb levels were those that presented the highest genetic damage. It has been reported that Pb generates indirect genotoxicity by increasing oxidative stress, producing ROS, and causing an imbalance in the redox state of the cell [70]; in cells subjected to chemical stress, the oxidation of macromolecules such as lipid peroxidation and oxidation of nitrogenous bases in the DNA is induced [71]. In addition, Pb causes indirect genotoxicity by replacing Zn in Zn finger proteins involved in DNA repair and replication mechanisms [72], which causes higher mutagenic rates and repair mistakes, DNA–protein crosslinks, and single- and double-strand breaks [73].

Early biomarkers of effect, such as the genotoxicity evaluated through the comet assay, have been used in several species of the Fabaceae family exposed to metals to determine their adverse biological effects. For example, in Trifolium repens, the higher bioaccumulation of Cd, Pb, Hg, Ni, and Zn resulted in significant genetic damage in leaf tissue cells [74]. In Vicia faba, a positive relationship was identified between Cr (VI) concentration and genetic damage in root cells [75]. T. repens exposed to Pb showed genetic damage in leaf tissue cells after three days of exposure [76] and, in Cassia occidentalis, a positive relationship was identified between the As, Ni, and Cr concentrations and genotoxicity in leaf tissue cells [77]. Similar results have been reported in the *P. laevigata* tree, where Pb bioaccumulation was positively related to genetic damage in leaf tissue [30]. Taking these results into account, we corroborate that Pb accumulation causes genetic damage (single-strand breaks, alkali-labile sites, and delayed repair sites) in *P. dulce* leaves, as reported in different species of the Fabaceae family. An interesting finding was that metal concentrations in *P. dulce* individuals established in the Cuautla River reflected the environmental contamination gradients identified in water and sediment samples. This is important because it further corroborates the usefulness of this tree species as a bioindicator of environmental quality for riparian ecosystems, in which there are no studies regarding biomonitoring strategies employing tree species.

Moreover, *P. dulce* meets the criteria for bioindicator species by having a wide distribution from 0 to 1800 m a.s.l. it is a dominant species that accumulates Cu, Pb, and Zn in leaves and bark, out of which, Pb causes genotoxic damage in leaf tissue. Despite the genetic damage caused by Pb, these individuals are tolerant to this metal, since they complete their phenological cycle and are established adults. Considering all these arguments, we can consider that *P. dulce* may serve as a bioindicator species for biomonitoring studies in riparian ecosystems.

## 4. Materials and Methods

### 4.1. Study Site: Cuautla River

The Cuautla River is geographically located between the parallels 18°30′ and 18°50′ N and 98°55′ and 99°10′ W in Morelos State, central Mexico [78]. It has a length of 110 km, covering an extension of 152.3 hectares. This site belongs to the Protected Natural Area known as “Los Sabinos, Santa Rosa, and San Cristóbal Springs.” In addition, this river is the home to many species of birds, mammals, amphibians, reptiles, and fishes. Among the most common tree species distributed along the Cuautla River is *P. dulce* [79]. *P. dulce,* commonly known as “Guamúchil”, belongs to the Fabaceae family; it is an evergreen tree with an average height of 15 to 20 m, which flowers from November to May, and the fruits ripen from March to August*. P. dulce* has been reported as a metal bioaccumulator species and is capable of translocating them to leaves and fruits [31,35,36,37].

### 4.2. Sampling Sites

In this study, six sampling sites along the Cuautla River were studied (S1–S6). These sites were chosen downstream of the river in the direction of the channel with a one-kilometer distance between each sampling site: S1 (0 m) was located on the slope of the spring called “El Almeal” (18°49′01.1″ N and 98°56′46.8″ W); S2 (1000 m) was located at coordinates 18°49′00.1″ N and 98°56′41.7″ W; S3 (2000 m) was located at 18°48′30.2″ N and 98°57′02.4″ W; S4 (3000 m) at 18°80′11.23″ N and 98°95′72.14″ W; S5 (4000 m) at 18°80′08.67″ N and 98°95′92.29″ W; and S6 (5000 m) at 18°46′45.1″ N and 98°58′24.1″ W.

**Figure 5:**
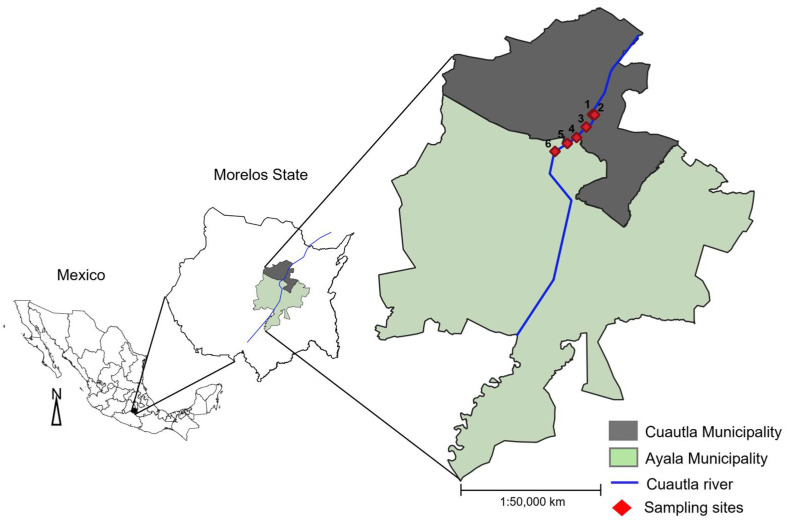
Study sites in Cuautla River, Morelos, Mexico.

The sampling sites were located downstream along the Cuautla River in areas with several wastewater discharge points (Figure 5). Various industries are located on the periphery of S1, including a soft drink production plant and automotive industries, as well as water extractive activities for industrial, domestic, and water purification purposes. In the surroundings of S2, automotive paint industries and domestic discharges are located. In the S3 area, there are hospitals, sports centers, shops, and domestic wastewater discharge points. In S4, several domestic discharge points were identified. A water treatment plant is located at S5; in the same area, farming activities and domestic wastewater discharges were also located. Finally, in the vicinity of S6, farming and livestock activities were identified.

### 4.3. Water and Sediment Sample Collection in the Cuautla River

Water and sediment samples were collected at each sampling site according to the Mexican standard NMX-AA-051-SCFI-2016 [80]. In total, 54 water and 54 sediment samples were obtained (9 water and 9 sediment samples per sampling site). The water samples were collected in 1L plastic containers previously washed with 70% nitric acid and rinsed three times with water from the study site, while the sediment samples were collected in plastic bags. All samples were transported to the Environmental Research Laboratory at the Biotechnology Research Center of the Autonomous University of Morelos State, water samples were kept at 4 °C, and the sediment samples were dried at room temperature until analyses.

### 4.4. Sampling of Leaf and Bark Tissue of P. dulce

For each sampling site, 10 adult individuals growing on the riverbank were randomly selected (n = 60). Leaves of the same age were sampled around the lower foliage (30 g of each sample) without apparent physical damage to assess genetic damage using the alkaline single-cell electrophoresis technique and metal quantification. Then, the leaf samples were placed in aluminum foil, stored in vacuum-sealed plastic bags, and transported at 4 °C to the Environmental Research Laboratory at the Biotechnology Research Center of the Autonomous University of Morelos State, where the samples were processed and analyzed. For bark metal determinations, whole bark tissue was taken from the external surface with a stainless-steel knife, splitting the inner part of the bark; the thickness of the samples was around 2 mm. Bark samples were taken from the stem at a height of 1.5–1.8 m above the ground from all sides of the trees. All the samples were stored in paper bags and labeled until later analysis.

### 4.5. Determination of Metal Concentration in Water and Sediment from the Cuautla River and in Leaf and Bark Samples of P. dulce

Metal concentration in water was analyzed in accordance with the provisions of the NMX-AA-051-SCFI-2016 Mexican standard [80]. At each sampling site, 9 samples (a total of 54 water samples) were analyzed, which were filtered with a 0.25 µm filter, and adjusted to pH equal to 1 with HNO_3_ for subsequent metal quantification. For the analysis of the sediment, 54 samples (9 samples at each sampling site were analyzed) were dried at room temperature, sieved with a 2 mm sieve, and then 0.25 g of each sample was weighed for subsequent metal quantification.

For the analysis of metal content in leaf and bark tissue of *P. dulce*, samples of six individuals randomly selected from each site (n = 36) were analyzed. The tissues were washed and dried at 50 °C for 72 h and ground and sieved to 2 mm size, and then 0.25 g of dry tissue of each sample was weighed. The acidic digestion for sediment and plant tissue samples was conducted in a microwave for accelerated reaction system (CEM^®^ MARS-5, CEM, Matthews, NC, USA) by adding 10 mL of concentrated HNO_3_; digestion was carried out in two 5 min cycles at 175 °C at 5 rpm and one 15 min cycle (50 °C) for the cooling phase. Once the digestion was completed, the samples were allowed to cool at room temperature, filtered, and filled with tetra-distilled water to a final volume of 50 mL.

Metal concentrations (Cd, Cr, Cu, Ni, Pb, and Zn) in environmental matrices and plant tissues were analyzed by an atomic absorption spectrophotometer (GBC 908 AA, Scientific Equipment Pty Ltd., Dandenog, Victoria, Australia). The detection limits in mg/L were Cd (0.0004), Cr (0.003), Cu (0.001), Ni (0.009), Pb (0.01), and Zn (0.0005). Determinations were conducted by triplicate for each water sample (mg/L) and those of sediment and plant tissues (mg/kg); the average values were reported as the total concentration for each metal in samples.

### 4.6. Evaluation of Genetic Damage in Leaf Tissue of P. dulce

To evaluate genotoxic damage in *P. dulce*, 10 individuals were randomly selected from each sampling site (n = 60), young leaf tissue of each individual was collected, and the alkaline single-cell electrophoresis technique or “Comet assay” was performed according to the protocol of Navarrete et al. [81] and Tice et al. [82] with modifications for plants [83]. The procedure was conducted under dark conditions at 4 °C. Leaves were washed with distilled water and dried. Two leaves of *P. dulce* from each individual were placed in 1 mL PBS buffer (1X, pH 7.4) at 4 °C, cut in parallel with a scalpel to release the cell nuclei, and left to rest for 5 min until the nuclei precipitated. Glass slides were used with a homogeneous layer of 1% regular melting-point agarose. Subsequently, 50 μL of the nuclei suspension in PBS was taken and mixed with 50 μL of 1% low-melting-point agarose, and 80 μL of the mixture was taken and spread on the slide previously treated with agarose. Subsequently, 80 μL of 0.5% low-melting-point agarose was placed on the slide and incubated for 5 min at 4 °C. Slides were placed in lysis solution (0.1 mol/dm^3^ Na_2_EDTA, 2.5 mol/dm^3^ NaCl, 0.01 mol/dm^3^ Tris, pH 10, 10% DMSO, and 1% Triton X-100) and stored at 4 °C until electrophoresis process.

During alkaline electrophoresis, 1.8 L of pH > 13 electrophoresis buffer (0.3 mol/dm^3^ NaOH, 1 mM EDTA) was placed in a horizontal electrophoresis chamber and incubated for 15 min to allow for the unwinding of the DNA strands. Electrophoresis was carried out at 25V, 300 mA for 20 min, and, finally, three washes were completed with 0.4 mol/dm^3^ Tris solution (pH 7.5) at 4 °C for neutralization, followed by fixation with 90% ethanol for 15 min. After that, the slides were dried and stained with 25 μL of ethidium bromide. The slides were read using a fluorescence microscope (Nikon Ni-SSR, 930806, Tokyo, Japan) at 590 nm. The tail length (DNA migration) of 100 cells for each *P. dulce* individual sample was measured using a scale integrated into the microscope, and the average tail length (indicative of the genotoxic damage) of 100 cells from each individual was reported in micrometers.

### 4.7. Statistical Analysis

Statistical analyses were carried out using STATISTICA 8 [84]. First, the normality of the experimental data was assessed using the Shapiro–Wilk test. Subsequently, linear regressions were conducted to determine the relationship between metal concentration in water and sediment and the sampling distance, and with the metal concentration in *P. dulce* leaves and bark tissue. Also, a linear regressions analysis was conducted to determine the relationship between metal content in *P. dulce* leaves and the genetic damage observed.

In addition, two-way analyses of variance (ANOVA) were conducted to determine the effect of the study site and the tissue (leaf and bark tissue) on metal concentration in *P. dulce* individuals. Subsequently, the analyses of variance were subjected to Tukey post-hoc tests to identify significant differences between tissues of individuals from the same site.

## 5. Conclusions

In this study, Cd and Pb were found in water samples, while Cd, Cu, Pb, and Zn were found in sediment samples of the Cuautla River. Likewise, this study showed that individuals of *P. dulce* established along the Cuautla River showed high Cu, Pb, and Zn concentrations in leaves and bark, with greater concentrations in leaf tissue than in bark. Copper levels in leaves of *P. dulce* and its concentration in Cuautla River sediments evidenced an increasing contamination gradient for this metal along the river channel. Moreover, according to Pb concentration in leaves and bark of *P. dulce* and Pb concentrations in water and sediment samples, a decreasing contamination gradient for Pb was also observed along the river channel. The analysis of genetic damage revealed that, as Pb concentration in leaves increases, the genetic damage levels also increase. Despite the registered Pb concentration and negative biological effects, *P. dulce* is a tree species that develops and survives in this riparian ecosystem contaminated with metals. Moreover, the metal concentrations registered in *P. dulce* individuals established in the Cuautla River reflected the environmental contamination gradients identified in water and sediment samples. Hence, *P. dulce* is a candidate species to serve as a bioindicator for biomonitoring studies to assess acute (leaf tissue) and chronic (bark) exposure to metals in riparian ecosystems in which there are no studies of biomonitoring strategies using tree species.

## Figures and Tables

**Table 1:** Metal concentration in water samples from the Cuautla River.

Metal	Sampling Site						NOM-127-SSA1-2021	WHO
	S1	S2	S3	S4	S5	S6		
	Concentration (mg/L) *							
Cd	**0.006 ± 0.001**	UDL	UDL	UDL	UDL	UDL	0.005	0.003
Pb	**0.509 ± 0.007**	**0.136 ± 0.010**	**0.15 ± 0.003**	**0.141 ± 0.007**	**0.311 ± 0.093**	**0.102 ± 0.004**	0.01	0.01

* Average ± SE (standard error). Numbers in bold indicate values higher than the maximum permissible limits stablish on the NOM-127-SSA1-2021 Mexican standard and the WHO (World Health Organization) guidelines. Cu, Cr, Ni, and Zn were not detected. UDL: under detection limits.

**Table 2:** Metal concentration in sediment samples from the Cuautla River.

Metal	Sampling Site						CCME, ISQG	CCME, PEL
	S1	S2	S3	S4	S5	S6		
	Concentration (mg/kg) *							
Cu	45.9 ± 1.4	UDL	UDL	UDL	UDL	UDL	35.7	197.0
Cd	**89.5 ± 39.1**	UDL	UDL	UDL	UDL	UDL	0.6	3.5
Pb	**427.0 ± 177.8**	**56.0 ±** **1.6**	**55.8 ±** **1.5**	**55.0 ± 5.2**	34.8 ± 1.7	26.2 ± 1.7	35.0	91.3
Zn	25.2 ± 4.8	22.6 ± 8.8	49.5 ± 17.3	83.1 ± 22.7	55.2 ± 21.6	103.2 ± 82.2	123.0	315.0

* Average ± SE (standard error). Numbers in bold indicate values higher than the maximum permissible limits stablish by the Canadian Council of Ministers of the Environment (CCME)’s interim sediment quality guideline (ISQG) and probable effects levels (PEL). Cr and Zn were not detected. UDL: under detection limits.

**Table 3:** Average concentration (±standard error) of metals in leaf tissue and bark of *P. dulce* individuals from each sampling site.

Metal	Sampling Site					
	S1	S2	S3	S4	S5	S6
	Concentration (mg/kg) *					
Leaf						
Cu	32.0 ± 0.8	25.4 ± 1.2	30.7 ± 0.6	35.2 ± 2.2	40.8 ± 1.4	44.6 ± 1.8
Pb	62.8 ± 6.9	41.6 ± 5.9	10.3 ± 2.1	10.8 ± 2.1	17 ± 4.1	7.9 ± 1.8
Zn	14.8 ± 2.1	27.7 ± 7.9	13.4 ± 2.4	6.3 ± 1.0	9.2 ± 2.6	3.2 ± 0.4
Bark						
Cu	9.6 ± 2.0	2.6 ± 0.5	12.1 ± 1.9	3.1 ± 0.5	13.4 ± 0.6	2.3 ± 0.8
Pb	37.6 ± 4.0	33.4 ± 1.4	26.6 ± 3.1	17.9 ± 2.9	11.9 ± 2.4	20.9 ± 2.8
Zn	14.2 ± 2.4	11.9 ± 1.5	9.1 ± 0.9	14.6 ± 2.3	15.7 ± 5.1	11.8 ± 4.1

* Average ± SE (standard error).

## Data Availability

Please contact author for data request.
